# Combined transarterial iodized oil injection and computed tomography-guided thermal ablation for hepatocellular carcinoma: utility of the iodized oil retention pattern

**DOI:** 10.1007/s00261-021-03305-3

**Published:** 2021-10-12

**Authors:** Jie Tan, René Michael Mathy, De-Hua Chang, Tian Tang, Zi-Shu Zhang, Yu-Dong Xiao

**Affiliations:** 1 Department of Radiology, The Second Xiangya Hospital of Central South University, No. 139 Middle Renmin Road, Changsha, 410011 China; 2 Department of Diagnostic and Interventional Radiology, University Hospital Heidelberg, 69120 Heidelberg, Germany; 3 Department of Interventional Radiology, The Affiliated Cancer Hospital of Xiangya School of Medicine, Central South University, Changsha, 410013 China

**Keywords:** Hepatocellular carcinoma, Transarterial chemoembolization, Ablation techniques, Matched-pair analysis, Survival analysis

## Abstract

**Purpose:**

To investigate whether the iodized oil (Lipiodol, Guerbet Group, Villepinte, France) retention pattern influences the treatment efficacy of combined transarterial Lipiodol injection (TLI) and thermal ablation in patients with hepatocellular carcinoma (HCC).

**Methods:**

Data of 198 patients (280 HCC lesions), who underwent TLI plus computed tomography (CT)-guided thermal ablation at three separate medical institutions between June 2014 and September 2020, were reviewed and analyzed. The Lipiodol retention pattern was classified as complete or incomplete based on non-enhanced CT at the time of ablation. The primary outcome was local recurrence-free survival (LRFS) for lesions; the secondary outcome was overall survival (OS) for patients. Propensity score matching (PSM) was performed using a caliper width of 0.1 between the two groups. Differences in LRFS and OS between the two groups were compared using the log-rank test.

**Results:**

A total of 133 lesions exhibited a complete Lipiodol retention pattern, while 147 exhibited an incomplete pattern. After PSM analysis of baseline characteristics of the lesions, 121 pairs of lesions were matched. LRFS was significantly longer for lesions exhibiting complete retention than for those exhibiting incomplete retention (*P* = 0.030). After PSM analysis of patient baseline characteristics, 74 pairs of patients were matched. There was no significant difference in OS between the two groups (*P* = 0.456).

**Conclusion:**

Lipiodol retention patterns may influence the treatment efficacy of combined TLI and thermal ablation for HCC lesions. However, a survival benefit for the Lipiodol retention pattern among HCC patients was not observed and needs further confirmation.

## Introduction

Hepatocellular carcinoma (HCC) accounts for 90% of primary liver cancers and is the fourth most common cause of cancer-related death worldwide. The treatment strategy for HCC depends on multiple factors, including tumor stage, underlying liver condition, and performance status [1]. According to the Barcelona Clinic Liver Cancer (BCLC) staging system [2], ablation is recommended as an alternative treatment option for patients who are not suitable candidates for resection or liver transplantation. For lesions < 3 cm in size, ablation has demonstrated a therapeutic efficacy comparable to surgical resection [3]; however, for larger tumors, the efficacy of ablation is limited due to insufficient and disproportional ablation zones [4]. Various techniques, such as the combination of transarterial chemoembolization (TACE) and ablation, have been proposed to improve the treatment efficacy of ablation for larger tumors [5]. Generally, TACE may decrease hepatic artery inflow, which reduces the “heat-sink” effect, resulting in an extended ablation area [6]. In addition, tumor visualization can be improved by iodized oil (Lipiodol, Guerbet Group, Villepinte, France) retention when performing ablation under computed tomography (CT) guidance [7]. A previous study by Lee et al. [8] demonstrated that intra-tumoral Lipiodol accumulation achieved by TACE before ablation may contribute to reducing local tumor progression in intermediate-size (2–5 cm) HCC lesions after ablation. However, the ablation procedures described by Lee et al. were performed exclusively under ultrasound guidance, which cannot be used to fully elucidate the advantage(s) of Lipiodol retention in thermal ablation, especially in the improvement of tumor visualization on non-enhanced CT [9]. Therefore, the purpose of the present study was to confirm previous results in a multicenter population and emphasize that tumor visualization can be influenced by the Lipiodol retention pattern, thus influencing the treatment efficacy of thermal ablation.

## Materials and methods

### Patients

This multicenter, retrospective study was approved by the institutional review board of the three participating hospitals and adhered to the principles of the Helsinki Declaration. Due to the retrospective nature of the study and the use of anonymized patient data, requirements for informed written consent were waived.

**Fig. 1:**
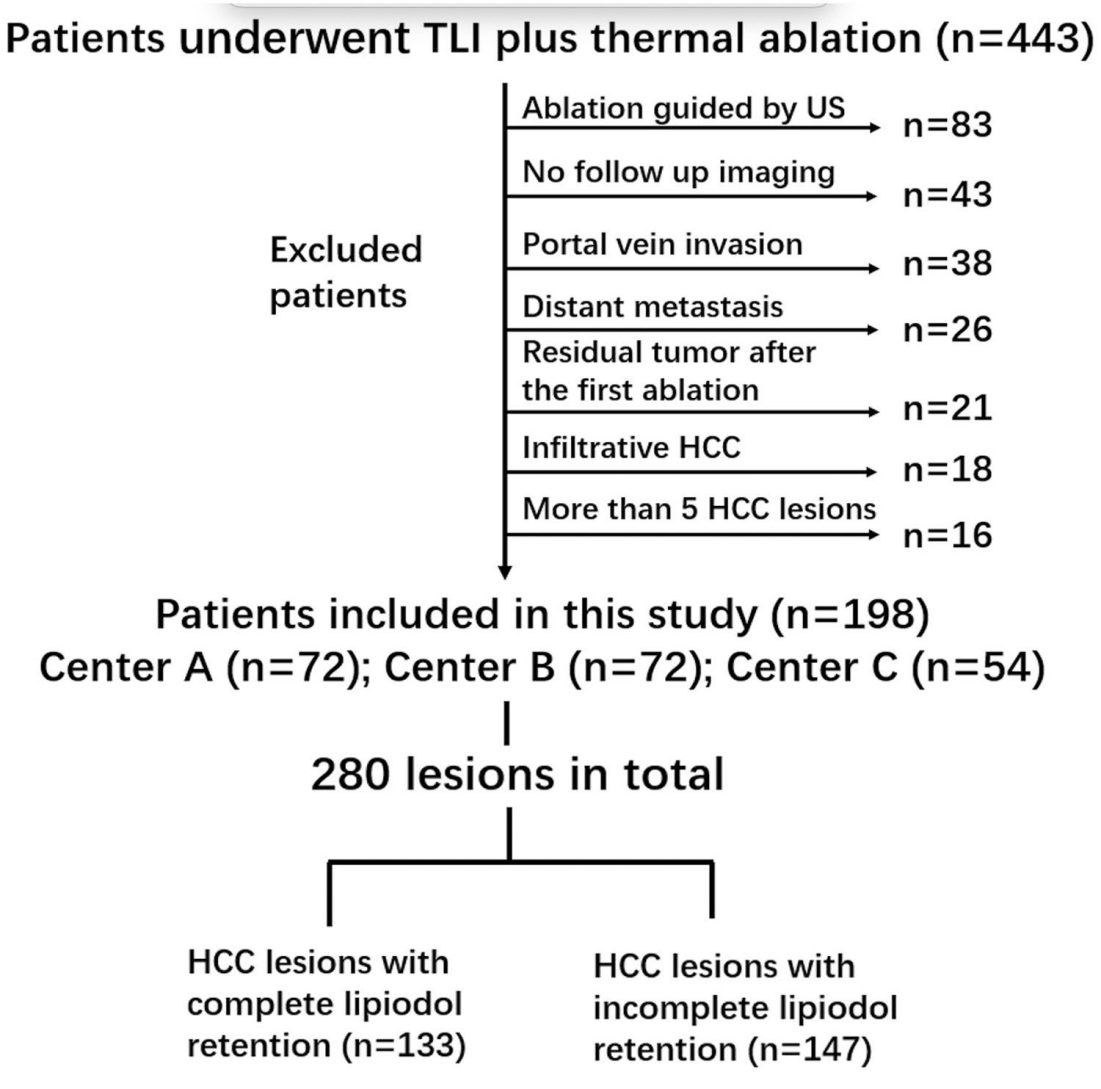
Flow diagram illustrating selection of the study population

Data of 443 consecutive patients with unresectable HCC, who underwent transarterial Lipiodol injection (TLI) plus thermal ablation at three separate institutions between June 2014 and September 2020, were collected, reviewed, and analyzed. One of the three institutions is in Germany, while the other two are located in China. Patients fulfilling the following criteria were included: preserved liver function (Child–Pugh A or B); Eastern Cooperative Oncology Group (i.e., ECOG) performance status 0; and at least one target lesion for measurement. Patients who underwent thermal ablation under ultrasound guidance (*n* = 83), those lost to follow-up (*n* = 43), individuals with macrovascular invasion (*n* = 38), distant metastasis (*n* = 26), residual tumor on contrast-enhanced CT or magnetic resonance imaging (MRI) ≤ 1 month after the first session of combination therapy (*n* = 21), infiltrative HCC (*n* = 18), and those with > 5 HCC lesions (*n* = 16) were excluded. Ultimately, 198 patients with 280 lesions were included in the present study. Among these 198 patients, 72 were from institution A, 72 were from institution B, and 54 were from institution C. A flow diagram illustrating the selection of the study population is presented in Fig. 1.

### TLI procedure

Treatment protocols for all patients were discussed at a multidisciplinary tumor board meeting. TLI was performed by several board-certified interventional radiologists. The femoral artery was routinely catheterized, and selective hepatic arteriography was performed. After hepatic arteriography, a coaxial microcatheter was placed as super-selectively as possible in the tumor feeders to slowly inject iodized oil (i.e., Lipiodol) alone or a Lipiodol–epirubicin/doxorubicin emulsion. The Lipiodol–epirubicin/doxorubicin emulsion was created by mixing up to 15 mL of Lipiodol and distilled water, and 50–120 mg of epirubicin or 100 mg of doxorubicin at a ratio of 3:1 or 2:1, respectively. In patients undergoing TACE, gelfoam slurry microspheres (Embozene Microspheres, Varian Medical System, Crawley, United Kingdom) or particles (PVA Embolization Particles, Cook Medical, Bloomington, Indiana, USA) were injected through the microcatheter to embolize the proximal tumor feeders, while in patients undergoing TLI alone, no additional chemotherapy or embolization was performed. All procedures were technically successful according to guidelines from the Society of Interventional Radiology [10].

### Thermal ablation procedure

Thermal ablation was performed using three microwave ablation (MWA) systems (KY-2000; Kangyou Medical Instrument Co. Ltd., Nanjing, China; Solero, AngioDynamics, Latham, NY, USA; Acculis, Balmer Medical SA, Concise, Switzerland) or two radiofrequency ablation (RFA) systems (Cool-tip, Covidien, Medtronic/Covidien, Dublin, Ireland; CELON Power System, Olympus Corporation, Tokyo, Japan) by several board-certified senior interventional radiologists. At the beginning of ablation, non-enhanced CT was performed, with the ablation antenna percutaneously inserted into the tumor under CT guidance. During ablation, the entire lesion with both the Lipiodol-marked—and, presumably not marked—portion was ablated as completely as possible. For tumors > 3.0 cm in size, an overlapping ablation technique was performed. For multifocal lesions, a maximum of three lesions was treated in a single session, and the remaining lesions were treated approximately 1 month after the first ablation session. MWA power was set to 60–140 W and the ablation time was 3–25 min; RFA power was set to 30–160 W and the ablation time was 10–40 min. Intraprocedural contrast-enhanced CT was performed to determine the ablative margin of the tumor. The technical success of ablation was defined as complete ablation of the tumor with a safety margin of at least 0.5 cm on CT images [11].

### Lipiodol retention pattern

**Fig. 2:**
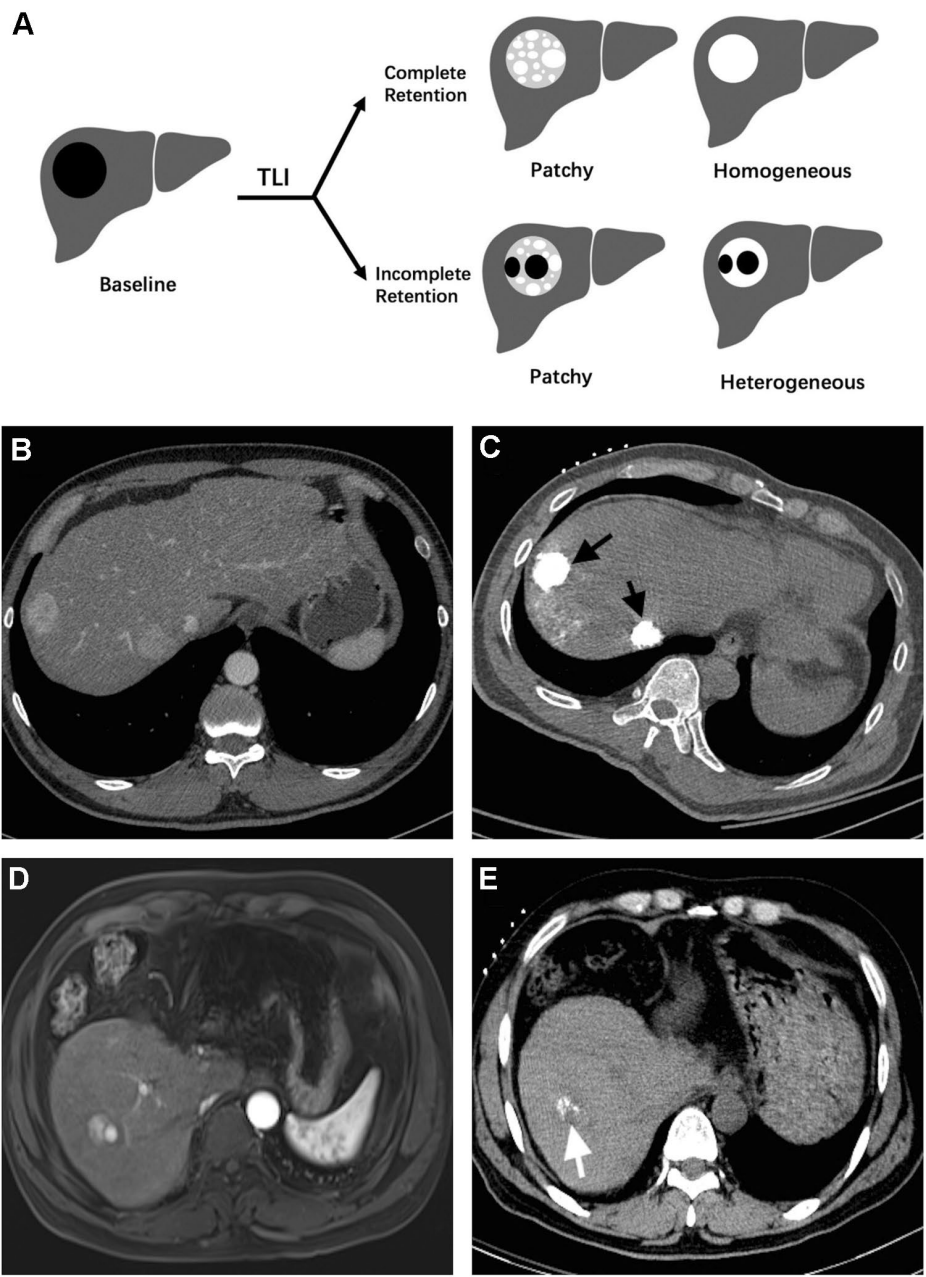
Schematic example and representative images of the complete and incomplete iodized oil (Lipiodol, Guerbet Group, Villepinte, France) retention patterns. Schematic representation of Lipiodol retention patterns on non-enhanced computed tomography (CT) (**A**). A 47-year-old male with two hypervascularized hepatocellular carcinoma (HCC) lesions in the right lobe of the liver (**B**). A homogeneous complete Lipiodol retention pattern after transarterial Lipiodol injection (TLI) is exhibited in two HCC lesions (black arrow), and the outlines of the lesions are well defined (**C**). A 50-year-old male with a hypervascularized HCC lesion in the right lobe of the liver (**D**). A heterogeneous incomplete Lipiodol retention pattern (white arrow) after TLI, and the outline of the lesion is poorly defined (**E**)

The Lipiodol retention pattern of each lesion was assessed on non-enhanced CT images at the time of ablation and classified into a complete retention pattern group and an incomplete retention pattern group. In a previous study [12], complete Lipiodol retention was defined as hyperattenuation of entire nodule volume compared to untreated liver parenchyma on post-TLI non-enhanced CT images (retention could be either homogenous or patchy, as long as the entire tumor volume exhibited Lipiodol retention), whereas incomplete Lipiodol retention was defined as the nodule volume exhibiting only partial hyperattenuation on post-TLI non-enhanced CT images. A schematic example and representative images of complete and incomplete Lipiodol retention patterns are presented in Fig. 2.

### Follow-up

After thermal ablation, patients were followed up at an interval of approximately 2 months in the first year and every 6 months in subsequent years. At each follow-up visit, contrast-enhanced CT/MRI of the liver was performed to evaluate local tumor recurrence (LTR) [13].

The primary outcome was local recurrence-free survival (LRFS) of the lesions; the secondary outcome was overall survival (OS) of the patients. LTR was defined as the appearance of tumor foci at the edge of the ablation zones after treatment beyond 1 month; however, the detection of viable tumor within 1 month after the first treatment session was defined as incomplete ablation, and such cases were excluded from the present study. LRFS was defined as the time interval between the first ablation and the LTR of the target lesion. OS was defined as the time interval between the first ablation and death from any cause or the last follow-up (March 7, 2021).

If tumor recurrence was identified, the choice of treatment modality for recurrent HCC was dependent on the site of the tumor, liver function, and the general condition of the patient.

### Data collection

Data for lesion-based variables were collected for each lesion, including the time interval between TLI and ablation (≤ 1, > 1 month), TLI alone or TACE, modality of thermal ablation (MWA or RFA), tumor size (≤ 3 cm, > 3 cm), watershed tumor (yes/no), perivascular location (yes/no), subcapsular location (yes/no), tumor shape (regular/irregular), and tumor vascularity (hypervascular/hypovascular). A watershed tumor was defined as a tumor involving ≥ 2 Couinaud–Bismuth segments of the liver [14]. Lesions with perivascular location were defined as tumors abutting the branches of a portal vein or hepatic vein with a lumen caliber ≥ 3 mm [13]. Lesions with subcapsular location were defined as target tumors located within 1 mm of the liver capsule [15]. Regular tumor shape was defined as a tumor with a smooth margin, presenting with a round or oval shape and an intact tumor capsule; otherwise, it was defined as an irregular shape [16]. A hypervascular tumor was defined as one that exhibited higher contrast enhancement than non-tumorous hepatic parenchyma in the arterial phase on pre-procedural CT/MRI; otherwise, it was defined as hypovascular [17].

Patient-based variables, including age, sex (male/female), number of tumors (single/multiple), Child–Pugh class (A/B), etiology of hepatitis (none, hepatitis B, hepatitis C, alcohol, others), cirrhosis (presence, absence), alpha-fetoprotein level (≤ 400 ng/mL, > 400 ng/mL), and platelet count, were collected for each patient.

### Statistical analysis

Categorical variables were compared using the χ^2^ test or Fisher’s exact test, as appropriate; continuous variables were compared using the Mann–Whitney *U* test or *t* test, as appropriate. To mitigate potential confounding and selection bias in the two groups, propensity score matching (PSM) was applied [18]. Each group was matched according to the generated PSM using a caliper width of 0.1. To compare the cumulative LRFS rate of lesions in the two groups, lesion-based variables were used to perform PSM analysis. To compare the OS rate for patients in the two groups, patient- and lesion-based variables were used to perform PSM analysis. The characteristics of the largest lesions in patients with multiple lesions were recorded for analysis. LRFS and OS in the two groups were estimated using the Kaplan–Meier method and compared using the log-rank test. Differences with a two-sided *P* < 0.05 were considered to be statistically significant. Statistical analysis was performed using SPSS version 24 (IBM Corporation, Armonk, NY, USA) or R software version 4.0.2 (http://www.R-project.org).

## Results

### Baseline patient characteristics

The entire study population included 161 males and 37 females, with a mean (± SD) age of 58.7 ± 11.3 years (range, 27–83 years). There were 123 patients with hepatitis B, 20 with hepatitis C, 15 with alcoholic hepatitis, and 19 with other etiologies of hepatitis. The diagnosis of HCC was based on pathology (*n* = 27) or the 2018 version of the Liver Imaging Reporting and Data System (i.e., LI-RADS) criteria (*n* = 171). There were 166 patients with Child–Pugh class A and 32 with Child–Pugh class B. Among the entire study population, 52 patients were very early stage (BCLC-0), 102 were early stage (BCLC-A), and 44 were intermediate-stage (BCLC-B). Baseline characteristics of patients in the three institutions are summarized in Table 1.

**Table 1:** The baseline patient characteristics among three institutions

Characteristics	Overall (*n* = 198)	Institution A (*n* = 72)	Institution B (*n* = 72)	Institution C (*n* = 54)	*P*
Age	58.7 ± 11.3	56.8 ± 10.5	55.0 ± 11.6	66.2 ± 8.3	< 0.001
Gender					0.121
Male	161 (81.3%)	60 (83.3%)	62 (86.1%)	39 (72.2%)	
Female	37 (18.7%)	12 (16.7%)	10 (13.9%)	15 (17.8%)	
Number of tumors					0.01
Single	118 (59.6%)	50 (69.4%)	33 (45.8%)	35 (64.8%)	
Multiple	80 (40.4%)	22 (30.6%)	39 (54.2%)	19 (35.2%)	
BCLC stage					< 0.001
0	52 (26.3%)	14 (19.4%)	10 (13.9%)	28 (51.9%)	
A	102 (51.5%)	45 (62.5%)	38 (52.8%)	19 (35.2%)	
B	44 (22.2%)	13 (18.1%)	24 (33.3%)	7 (12.9%)	
Child–Pugh class					0.859
A	166 (83.8%)	61 (84.7%)	59 (81.9%)	46 (85.2%)	
B	32 (16.2%)	11 (15.3%)	13 (18.1%)	8 (14.8%)	
Etiologies of hepatitis					< 0.001
None	21 (10.6%)	16 (22.2%)	3 (4.2%)	2 (3.7%)	
HBV	123 (62.1%)	56 (77.8%)	63 (87.4%)	4 (7.4%)	
HCV	20 (10.1%)	0	3 (4.2%)	17 (31.5%)	
Alcohol	15 (7.6%)	0	2 (2.8%)	13 (24.1%)	
Others	19 (9.6%)	0	1 (1.4%)	18 (33.3%)	
Cirrhosis					< 0.001
Yes	148 (74.7%)	58 (80.6%)	39 (54.2%)	51 (94.4%)	
No	50 (25.3%)	14 (19.4%)	33 (45.8%)	3 (5.6%)	
AFP					0.003
≤ 400 ng/mL	167 (84.3%)	55 (76.4%)	59 (81.9%)	53 (98.1%)	
> 400 ng/mL	31 (15.7%)	17 (23.6%)	13 (18.1%)	1 (1.9%)	
Platelet	127.9 ± 74.5	118.2 ± 63.8	134.0 ± 63.9	132.6 ± 97.4	0.384

*BCLC* Barcelona-Clinic Liver Cancer, *AFP* Alpha fetoprotein

### Comparison of LRFS between complete and incomplete retention after one-to-one PSM

**Fig. 3:**
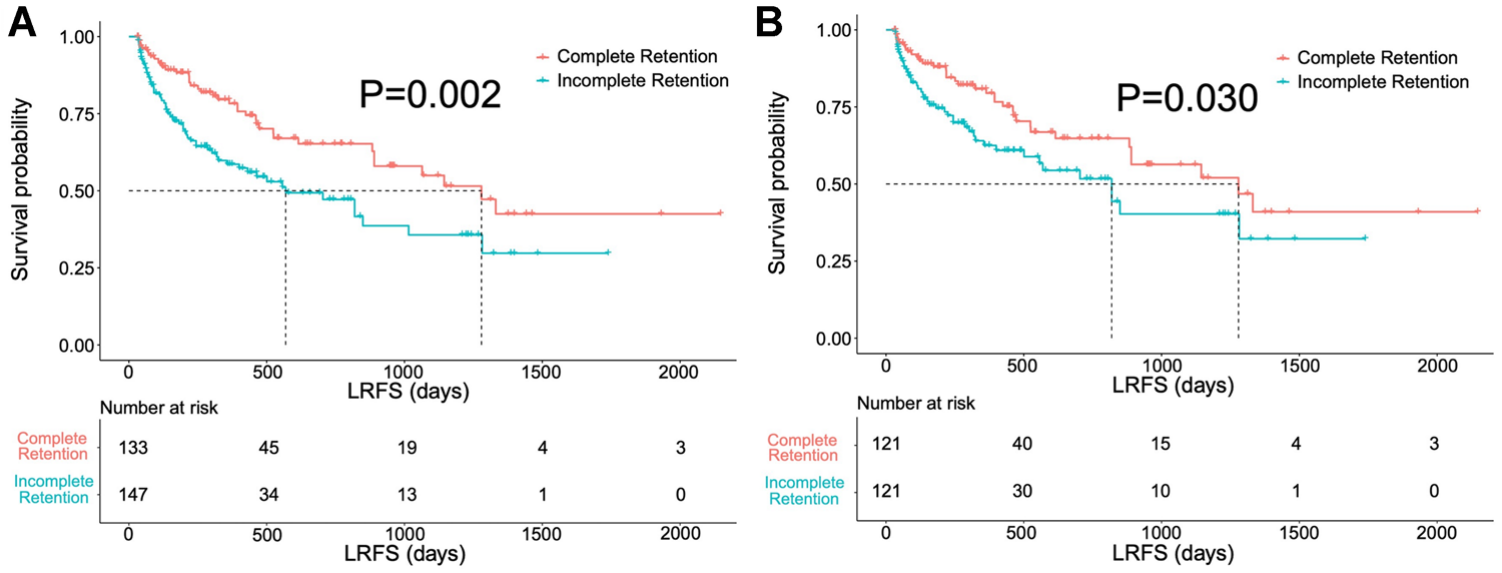
Kaplan–Meier LRFS curves for patients with complete and incomplete iodized oil (Lipiodol, Guerbet Group, Villepinte, France) retention patterns before and after propensity score matching (**A**) and (**B**), respectively

A total of 133 lesions comprised the complete retention group and 147 comprised the incomplete retention group before PSM. The median LRFS was 1279 days (95% confidence interval [CI] 856–1702 days) in the complete retention group and 569 days (95% CI 290–848 days) in the incomplete retention group before PSM. LRFS was significantly longer in the complete retention group than in the incomplete retention group (*P* = 0.002) (Fig. 3a). After one-to-one PSM, 121 pairs of lesions were matched (Table 2). The median LRFS was 1279 days (95% CI 821–1746 days) in the complete retention group and 819 days (95% CI 521–1117 days) in the incomplete retention group after PSM. LRFS was significantly longer in the complete retention group than in the incomplete retention group (*P* = 0.030) (Fig. 3b).

**Table 2:** Baseline characteristics of complete retention group and incomplete retention group for the comparison of LRFS before PSM and after PSM

Characteristics	Before PSM			After PSM		
	Complete retention (*n* = 133)	Incomplete retention (*n* = 147)	*P*	Complete retention (*n* = 121)	Incomplete retention (*n* = 121)	*P*
Time interval			0.001			0.117
Within 1 month	104	88		92	81	
Beyond 1 month	29	59		29	40	
TLI			0.480			0.649
Alone	27	35		27	30	
TACE	106	112		94	91	
Ablation type			0.819			0.497
MWA	109	122		98	102	
RFA	24	25		23	19	
Tumor size			0.008			0.253
≤ 3 cm	102	91		91	83	
> 3 cm	31	56		30	38	
Watershed			0.094			0.512
Yes	21	35		21	25	
No	112	112		100	96	
Perivascular			0.008			0.367
Yes	54	83		53	60	
No	79	64		68	61	
Subcapsular			0.708			0.691
Yes	87	93		77	74	
No	46	54		44	47	
Tumor shape			0.002			0.354
Regular	109	97		97	91	
Irregular	24	50		24	30	
Vascularity			0.108			0.307
Hyper-	113	134		105	110	
Hypo-	20	13		16	11	

*LRFS* Local recurrence-free survival, *PSM* Propensity score matching, *TLI* Transarterial Lipiodol injection, *MWA* Microwave ablation, *RFA* Radiofrequency ablation

### Comparison of LRFS between complete and incomplete retention subgroups

LRFS in the complete and incomplete Lipiodol retention groups were further analyzed in subgroups of tumor size (≤ 3 cm, > 3 cm), the time interval between TLI and ablation (within/beyond 1 month), lesions with perivascular location (yes/no), and lesions that underwent TACE or TLI alone.

**Fig. 4:**
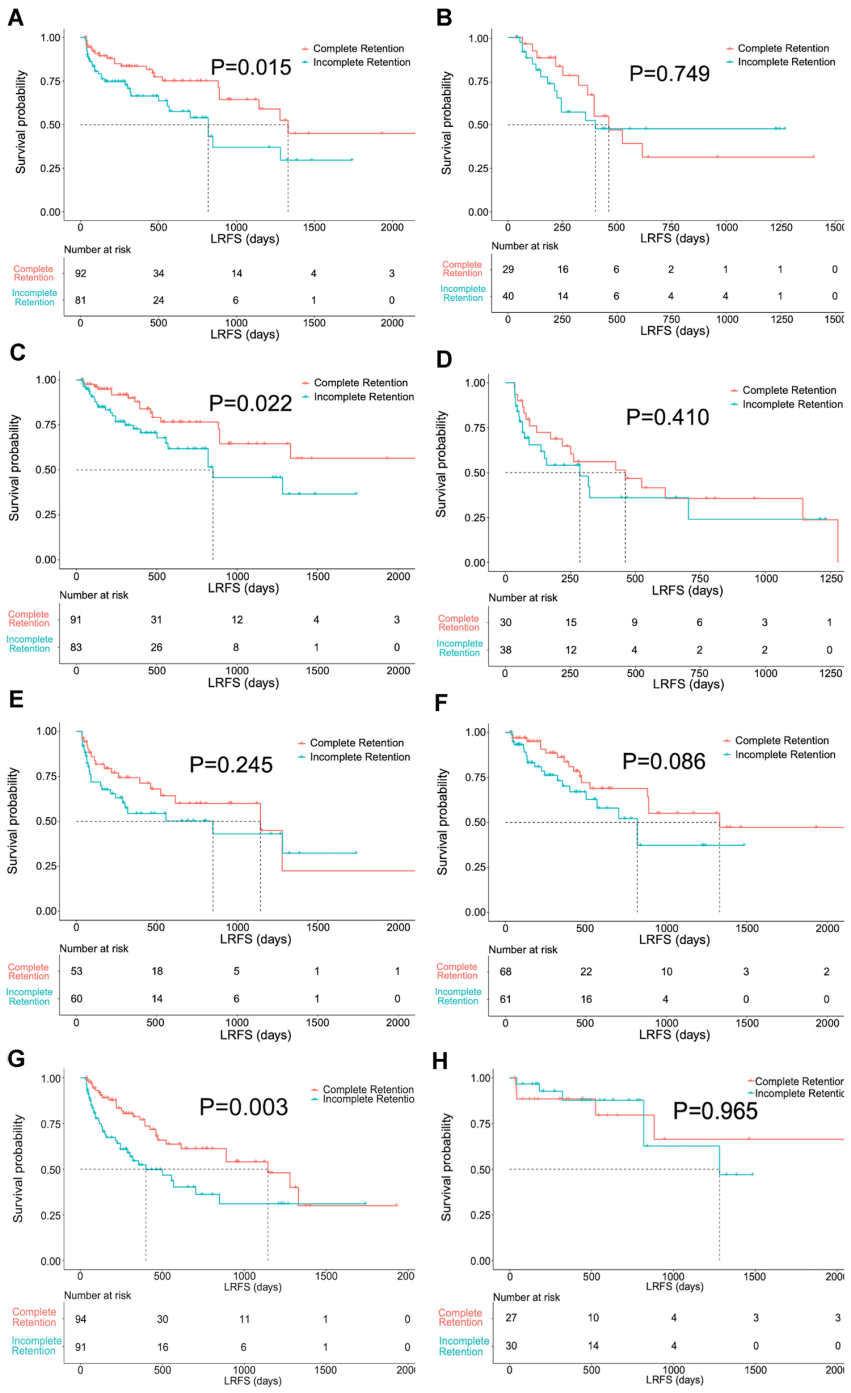
Kaplan–Meier LRFS curves for patients with complete and incomplete iodized oil (Lipiodol, Guerbet Group, Villepinte, France) retention in the subgroups with a time interval between transarterial Lipiodol injection (TLI) and ablation of within 1 month (**A**) or beyond 1 month (**B**), a tumor size < 3 cm (**C**) or > 3 cm (**D**), a perivascular (**E**) or non-perivascular (**F**) location of hepatocellular carcinoma (HCC) lesions, performed transarterial chemoembolization (TACE) (**G**) or TLI alone (**H**)

In the subgroup with a time interval between TLI and ablation of ≤ 1 month, lesions exhibiting complete Lipiodol retention demonstrated a better LRFS than those with incomplete retention (*P* = 0.015) (Fig. 4a). However, no difference was noted in the subgroup with a time interval between TLI and ablation > 1 month (*P* = 0.749) (Fig. 4b).

In the subgroup of lesions < 3 cm, those exhibiting complete Lipiodol retention demonstrated significantly better LRFS than lesions with incomplete retention (*P* = 0.022) (Fig. 4c). Nevertheless, there was no significant difference in the subgroup of lesions > 3 cm (*P* = 0.410) (Fig. 4d).

In the subgroup of lesions with and without perivascular location, both lesions with perivascular location (*P* = 0.245) (Fig. 4e) and without perivascular location (*P* = 0.086) (Fig. 4f) demonstrated no differences in LRFS between complete and incomplete Lipiodol retention.

In the subgroup of lesions treated with TACE plus thermal ablation, better LRFS was noted in lesions with complete Lipiodol retention than in those with incomplete Lipiodol retention (*P* = 0.003) (Fig. 4g). Nevertheless, there was no significant difference in the subgroup of lesions treated with TLI alone plus thermal ablation between complete Lipiodol retention and incomplete Lipiodol retention (*P* = 0.965) (Fig. 4h).

### Comparison of OS between complete and incomplete retention patterns

**Fig. 5:**
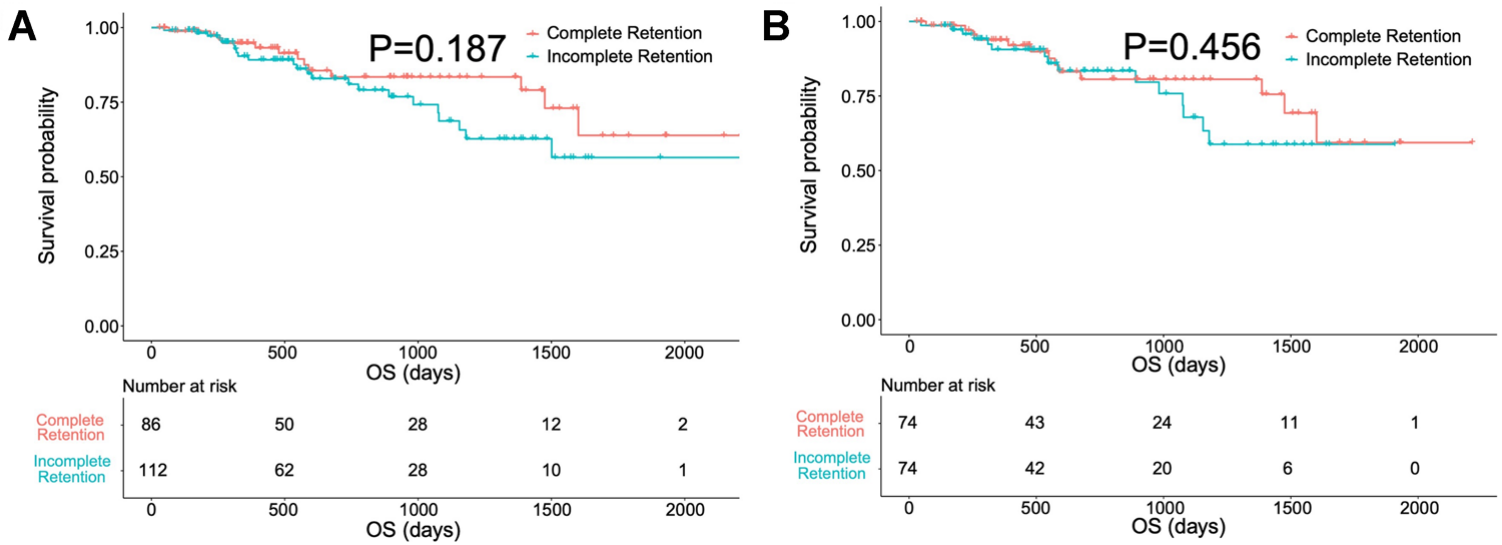
Kaplan–Meier OS curves for patients with complete and incomplete iodized oil (Lipiodol, Guerbet Group, Villepinte, France) retention before (**A**) and after (**B**) propensity score matching

Before PSM, 86 and 112 patients were classified into complete and incomplete Lipiodol retention groups, respectively. There was no difference in OS between the two groups (*P* = 0.187) (Fig. 5a). After one-to-one PSM, 74 pairs of patients were matched (Table 3). The median OS was not reached due to the short follow-up time. There was no difference in OS between the two groups (*P* = 0.456) (Fig. 5b).

**Table 3:** Baseline characteristics of complete retention group and incomplete retention group for the comparison of OS before PSM and after PSM

Characteristics	Before PSM			After PSM		
	Complete retention (*n* = 86)	Incomplete retention (*n* = 112)	*P*	Complete retention (*n* = 74)	Incomplete retention (*n* = 74)	*P*
Age	59.69 ± 11.13	57.99 ± 11.49	0.298	59.86 ± 11.23	59.09 ± 11.36	0.679
Gender			0.070			0.372
Male	65	96		60	64	
Female	21	16		14	10	
Number of tumors			0.510			0.865
Single	49	69		47	46	
Multiple	37	43		27	28	
Child–Pugh class			0.110			0.181
A	68	98		59	65	
B	18	14		15	9	
Etiologies of hepatitis			0.196			0.760
None	7	14		7	5	
HBV	54	69		43	49	
HCV	9	11		9	9	
Alcohol	10	7		9	5	
Others	6	11		6	6	
Cirrhosis			0.220			0.840
Yes	68	80		59	58	
No	18	32		15	16	
AFP			0.833			0.814
≤ 400 ng/mL	72	95		63	64	
> 400 ng/mL	14	17		11	10	
Platelet	122.49 ± 79.14	132.07 ± 70.74	0.371	125.05 ± 84.89	125.93 ± 70.91	0.946
Time interval			0.005			0.569
Within 1 month	69	69		57	54	
Beyond 1 month	17	43		17	20	
TLI			0.843			0.707
Alone	21	26		20	18	
TACE	65	86		54	56	
Ablation type			0.979			0.831
MWA	70	91		60	61	
RFA	16	21		14	13	
Tumor size			0.054			0.866
≤ 3 cm	57	59		46	45	
> 3 cm	29	53		28	29	
Watershed			0.243			0.711
Yes	19	33		19	21	
No	67	79		55	53	
Perivascular			0.111			0.742
Yes	41	68		38	40	
No	45	43		36	34	
Subcapsular			0.757			0.731
Yes	55	74		49	47	
No	31	38		25	27	
Tumor shape			0.012			0.351
Regular	68	70		57	52	
Irregular	18	42		17	22	
Vascularity			0.839			0.785
Hyper-	76	100		67	66	
Hypo-	10	12		7	8	

In patients with multiple lesions, the characteristics of the largest lesion were recorded for analysis

*OS* overall survival, *PSM* Propensity score matching, *AFP* Alpha fetoprotein, *TLI* transarterial Lipiodol injection, *MWA* Microwave ablation, *RFA* Radiofrequency ablation

## Discussion

Results of the present study revealed that HCC lesions exhibiting a complete Lipiodol retention pattern were associated with a significantly better LRFS than those with an incomplete pattern. This finding is consistent with other studies reporting that compact Lipiodol retention was associated with a lower risk for local progression after an initial TACE session, as well as in patients who underwent TACE followed by RFA [8, 12]. There is accumulating evidence that combination therapy with TACE and thermal ablation can result in better recurrence-free and OS than thermal ablation alone in patients with larger (> 3 cm) HCC tumors [19]. This synergistic effect is probably due to an increase in the thermal efficiency of ablation and the thermal sensitivity of tumor cells.

Results of the present study demonstrated a better LRFS in lesions with complete Lipiodol retention than in those with incomplete retention in the subgroup of lesions treated with TACE plus thermal ablation, whereas this difference was not observed in the subgroup of lesions treated with TLI alone plus thermal ablation. Usually, complete Lipiodol retention in a lesion reflects a greater deposition of chemotherapy drugs within the tumor than in a lesion with incomplete retention, which probably explains the difference in LRFS in this subgroup. However, for lesions that underwent TLI alone without the antitumor effect of chemotherapy drugs, this difference was not observed.

Interestingly, an essential finding of the present study was that LRFS was longer in lesions < 3 cm in size exhibiting a complete Lipiodol retention pattern than in those with incomplete retention, while this difference was not noted in tumors > 3 cm in size. Evidence suggests that thermal ablation alone can achieve complete tumor necrosis in tumors < 3 cm, which has demonstrated a treatment efficacy similar to that of surgical resection [3]. Therefore, the treatment efficacy of combination therapy of lesions < 3 cm with complete or incomplete Lipiodol retention patterns should, theoretically, be the same. However, the present study yielded the opposite finding. One possible reason for this discrepancy may be that a small lesion with an incomplete Lipiodol retention pattern is often associated with relatively poor visibility on non-enhanced CT imaging [7]. An additional explanation for this is probably that super-selective catheterization of small lesions was often not possible because the caliber of the feeding artery was too small. Instead, only segmental TLI could be performed; as such, Lipiodol also accumulated in the surrounding liver parenchyma, reducing the visibility of the lesion. Therefore, the outline of small tumors may have not been clearly displayed, which may have led to misplacement of the ablation needle under CT guidance, resulting in an inadequate safety margin. For tumors > 3 cm, the Lipiodol retention pattern did not have a significant influence on LRFS. It is possible that due to the better visibility of larger lesions compared with smaller lesions, needle misplacement could be avoided. Another possible reason is that large HCCs are more often heterogeneously hypervascularized, whereas this is rarely the case in small HCC lesions. Therefore, in small HCCs, the non-Lipiodol-retaining portions were probably not treated sufficiently, and the portion without retention remained active. In large HCCs, non-Lipiodol-retaining tumor portions are primarily hypovascularized and are less active. In summary, on the one hand, the results suggest that in the more poorly visualizable small lesions with incomplete Lipiodol retention, previous TACE as a supporter of the ablation was important to avoid an inadequate safety margin and/or to act as a “safety net” in case of an inadequate safety margin than in larger ones and, therefore, complete Lipiodol retention was important in these cases. On the other hand, incomplete Lipiodol retention in small lesions is more likely an indicator of untreated active tumor regions, whereas in larger lesions, this is only an expression of heterogeneous tumor vascularization.

Additionally, results of the present study demonstrated that lesions exhibiting a complete Lipiodol retention pattern were associated with significantly longer LRFS than those with an incomplete retention pattern if ablation was performed ≤ 1 month after the TLI procedure, while this difference was not found if ablation was performed > 1 month after TLI. It has been established that complete Lipiodol retention implies a more thorough reduction in tumor vascularity, which can reduce the tissue cooling effect and potentiate the efficacy of thermal ablation [20]. However, revascularization of the tumor and Lipiodol washout may occur over time, which may amplify the tissue cooling effect, thus reducing the treatment efficacy of ablation, even in lesions with complete Lipiodol retention [6, 21, 22]. As previously described, the heat-sink effect is more common in lesions with perivascular location, and embolization of the tumor-feeding arteries may reduce the heat-sink effect [4, 8, 21]. Therefore, both subgroups of lesions—perivascular and non-perivascular location—should, theoretically, exhibit better improvement after ablation for lesions with complete Lipiodol retention than for those with incomplete retention. However, the results of the present study do not support this finding. A probable explanation for this phenomenon is that the primary thermal ablation modality used in the present study was MWA [82.5% (231/280)], and several previous studies have demonstrated that MWA is less affected by the heat-sink effect than RFA [23].

The present study failed to demonstrate a significant difference in OS between patients with complete Lipiodol retention and those with incomplete retention. The OS of HCC patients is related to numerous factors, including age, liver function, severity of the underlying liver disease, tumor burden, antiviral therapy, and performance status [24–28]. Although the Lipiodol retention pattern may play an important role in the treatment efficacy of combination therapy for HCC lesions, this factor is not sufficient to influence the OS of HCC patients.

There were several limitations to the present study: the first of which was its multicenter, retrospective design and the inevitable heterogeneity of treatments (i.e., three centers, different ablation devices, and different chemotherapeutic and embolic agents) among institutions, which may have introduced bias. Although PSM was used to mitigate potential confounding and selection bias, some heterogeneities between the two groups persisted, such as TLI alone/TACE and the time interval of TLI and ablation. Second, the limited follow-up period of the present study may have led to a lack of thorough survival assessment among patients. Finally, the sample size used in the present study was relatively small; as such, a prospective study with a larger sample size is required.

In conclusion, lesions exhibiting a complete Lipiodol retention pattern demonstrated a better LRFS than those with an incomplete retention pattern, especially if the lesions were < 3 cm in size, a shorter time interval between TLI and ablation was chosen, and lesions were treated with TACE plus ablation. However, a survival benefit of the complete Lipiodol retention pattern for HCC patients was not observed and needs further confirmation.

## Data Availability

The datasets used and/or analyzed during the current study are available from the corresponding author on reasonable request.
